# Experimental evidence for social learning in semi-natural, wild-type Norway rats

**DOI:** 10.1038/s41598-025-25316-6

**Published:** 2025-10-27

**Authors:** Sacha C. Engelhardt, Harshkumar Vasoya, Michael Taborsky

**Affiliations:** 1 Department of Sociobiology and Anthropology, Johann-Friedrich-Blumenbach Institute for Zoology and Anthropology, University of Göttingen, 37077 Göttingen, Germany; 2 Behavioural Ecology and Sociobiology Unit, German Primate Center, Leibniz Institute for Primate Research, 37077 Göttingen, Germany; 3 Behavioural Ecology, Institute of Ecology and Evolution, University of Bern, 3032 Hinterkappelen, Switzerland; 4 Animal Minds Group, Institute of Neuroscience, Universitat Autònoma de Barcelona, Bellaterra, Barcelona, 08193 Spain; 5 Department of Collective Behavior, Max Planck Institute of Animal Behavior, 78467 Konstanz, Baden-Württemberg, Germany

**Keywords:** Cognition, Information acquisition, Innovation, Learning, Social transmission, Ecology, Ecology, Evolution, Neuroscience, Psychology, Psychology, Zoology

## Abstract

Animals may acquire information about their environment by social learning. Social transmission can affect the rate of trait acquisition and performance. It is often unclear, how behaviours are acquired when social information is available. In particular, the role of social learning in the acquisition of non-intuitive tasks is currently obscure. We asked whether wild-type Norway rats, *Rattus norvegicus*, in six semi-natural outside colonies benefit from each other in the acquisition and performance of a non-intuitive task by social learning. The task involved the opening of a seesaw mechanism to obtain a food reward. We induced innovations in four of six colonies and controlled the number of trained individuals and the relatedness composition. The latency to the first successful seesaw manipulation was shorter for naïve rats living with four experienced rats than for those living with zero or two experienced rats. The intervals between successful seesaw manipulations were not affected by the number of experienced rats and the colonies’ relatedness composition. Rats in four colonies innovated seesaw manipulations by trial-and-error learning. Our data show that information about the solution of a non-intuitive food acquiring problem can be socially transmitted among wild-type Norway rats, irrespective of the colonies’ relatedness composition.

**Supplementary Information:**

The online version contains supplementary material available at 10.1038/s41598-025-25316-6.

## Introduction

Social cognition, which includes social learning, allows animals to acquire and use information about their environment by means of social partners^1,2^. The social learning hypothesis proposes that the acquisition of a novel behavioural trait is facilitated by observing, or interacting with, individuals or their products^2,3^. Social learning is widespread among animal taxa^1,2^, facilitating adaptive decision-making to cope with ecological demands^4,5^. Social transmission is a subset of social learning affecting the rate at which an individual (i) acquires a novel trait, and (ii) performs it once acquired^2^. Social transmission is defined as occurring when the prior acquisition of a behavioral trait T by one individual A, when expressed either directly in the performance of T or in some other behavior associated with T, exerts a lasting positive causal influence on the rate at which another individual B acquires and/or performs T^2^. The demonstrator-observer pairing with controls (e.g. without a demonstrator) is a traditional experimental design for social learning^2,3^, and the latency to acquire an ability typically decreases as the number of experienced individuals increases^6–8^. The effect of living with different numbers of experienced individuals in a natural setting on the rates of acquisition and performance of skills has yet to be assessed.

The probability of learning socially may be proportional to the coefficient of relatedness between individuals^2,9^. Vertical transmission of socially learned traits from mother to offspring has been shown in non-human primates^9–11^ and in black bears, *Ursus americanus*^12^, but not in wild white-faced capuchin monkeys, *Cebus capucinus*, and wild vervet monkeys, *Chlorocebus pygerythrus*^13,14^. Social learning of food preferences in Norway rats, *Rattus norvegicus*, was independent of relatedness and familiarity in the laboratory^15,16^. An assessment of relatedness effects on social learning within groups of related or unrelated social partners under natural conditions seems to be called for.

A new or modified behaviour not previously found in the population is an innovation^2,17^. An innovation can spread in a population by social and asocial learning^17–20^. Social learning is often initiated by one individual’s innovation through using trial-and-error learning while interacting with the physical environment, based on personal information^21^. Naïve individuals, especially those living without experienced ones, should be more likely to innovate than experienced individuals, since experienced ones that had acquired the trait in the past can already successfully accomplish the goal, whereas naïve individuals living without experienced ones have to innovate new behaviours to succeed.

**Fig. 1:**
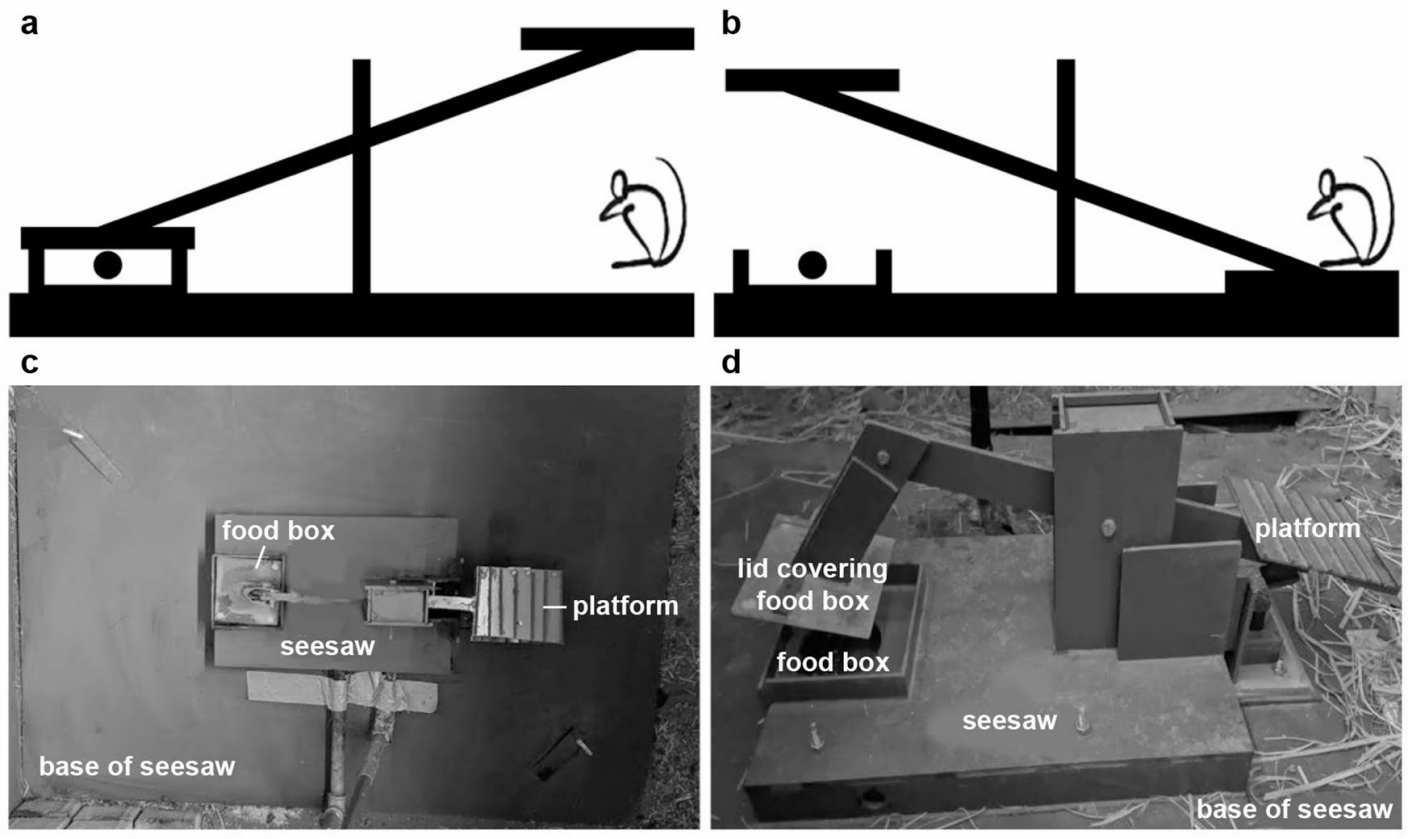
The seesaw. **(a**) Seesaw closed. The lid covers the food box, i.e. food rewards cannot be accessed. (**b**) Seesaw opened. By sitting on the platform, the rat lowers the platform and raises the lid, i.e. food rewards are accessible. (**c**) The seesaw’s platform, food box and base seen from above. (**d**) The seesaw’s platform before it connects with the electromagnet and microswitches located under it. When the position of the seesaw was changed by moving the seesaw to a different location inside the enclosures to account for local enhancement, the platform and food box’s positions were inversed.

The aim of this study was to test the social transmission of information about a complex task by social learning in a highly social animal in a semi-natural setting. Individual wild-type Norway rats asocially learned how to open the lid of a food container by sitting on a platform of a seesaw (Fig. 1), thereby creating “experienced rats”. We introduced such experienced rats to four of six colonies. We controlled (i) the number of individuals trained to manipulate a food provisioning mechanism, a seesaw test^22^, and (ii) the relatedness composition of colonies. To assess the hypothesis that wild-type Norway rats housed in semi-natural colonies acquire and perform a non-intuitive food provisioning task by social learning, we predicted that (1) the intermediate steps prior to first success (i.e. attending the platform or eating food rewards when another rat lowered the platform, thereby witnessing that conspecifics successfully manipulate the seesaw) should occur more often for naïve rats living with more experienced rats, (2) the latency to the first successful manipulation of the seesaw should be shorter for naïve rats living with more experienced rats, and (3) the intervals between successful manipulations affecting the rate of trait performance once acquired should be shorter for naïve rats as the number of experienced rats increases. To test the hypothesis that relatedness between individuals improves the acquisition and performance of a difficult food provisioning task during social learning, we predicted that: (4) the intermediate steps prior to first success should occur more often, the latency to the first successful manipulation of the seesaw should be shorter, and the intervals between successful manipulations should be shorter for naïve rats living in colonies with a higher relatedness composition. (5) We predicted that the likelihood of successful innovated manipulations should be greater for naïve rats than for experienced rats. A rat will have innovated a behaviour to successfully manipulate the seesaw if the behaviour is a new behaviour (e.g. directly lifting the lid of the seesaw rather than sitting on the platform, which is at the opposite end of the lid, or sitting on the platform in colonies without experienced rats who would have shown this behaviour) or a modified behaviour (i.e. a naïve or experienced rat directly lifting the lid of the seesaw instead of sitting on the platform in colonies with experienced rats).

## Results

**Fig. 2:**
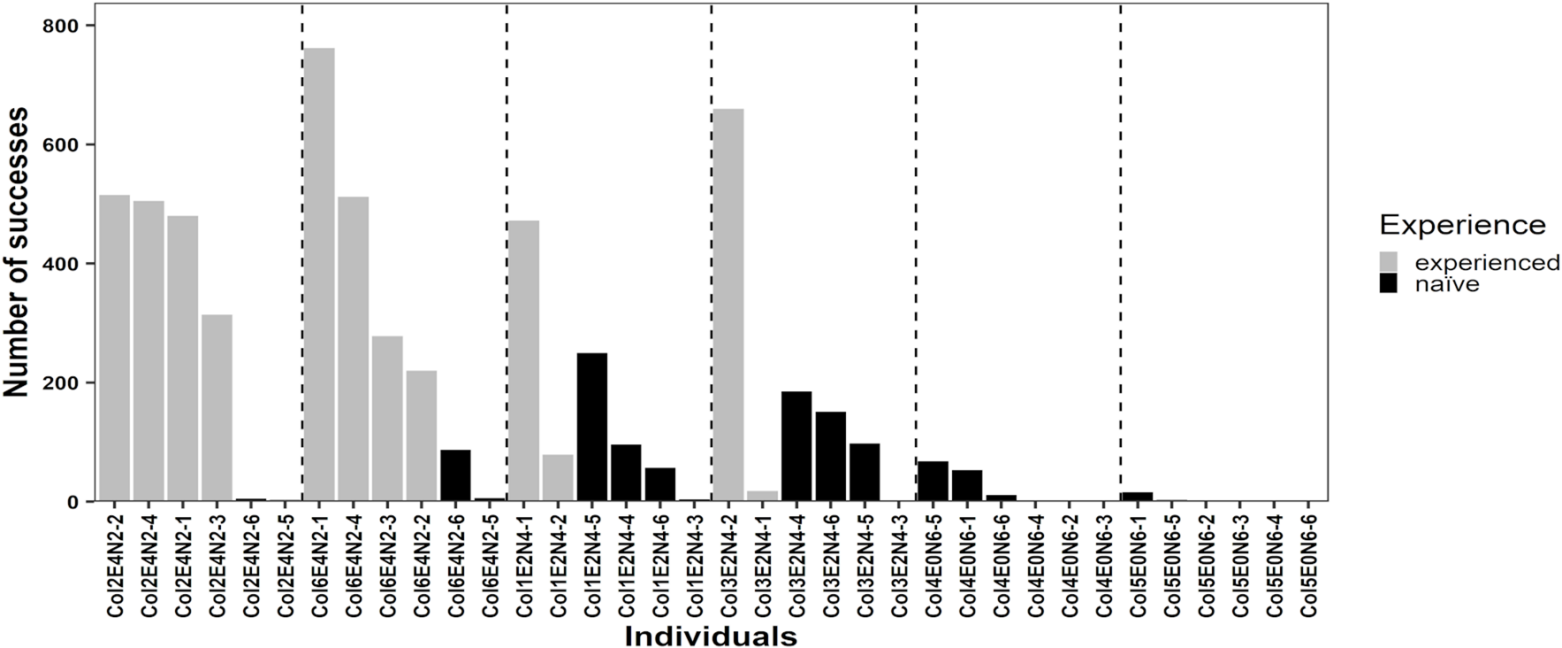
The cumulative number of successful seesaw openings to access food by experienced and naïve rats. The figure is ordered by the decreasing number of experienced rats by enclosure, by the decreasing relatedness composition, by the experience state of rats, i.e. experienced rats first, and finally by the decreasing number of successes. Individual identities on the abscissa are denoting the colony number, the number of experienced rats in the colony, the number of naïve rats in the colony, and the rat identity from 1 to 6. For example, “Col1E2N4-1” is read as *Col1* refers to colony 1, *E2* refers to 2 experienced rats in the colony, *N4* refer to 4 naïve rats in the colony, *−1* refers to rat No. 1. ‘Experience’ in the legend title refers to experienced rats (grey bars) and naïve rats (black bars). The dotted lines separate colonies.

Experienced rats were trained to acquire the non-intuitive food provisioning task prior to the main project, and the experienced rats acquired the trait after 19 to 26 training sessions. Seventeen of 24 naïve rats (70.8%) successfully manipulated the seesaw to access a food reward. In the experiment, six of the unsuccessful naïve rats lived in colonies without experienced rats, and the seventh unsuccessful naïve rat lived in a colony with 2 experienced rats (Fig. 2). Twenty-three out of 24 (95.8%) naïve rats sat on the platform, and the naïve rat who did not sit on the platform was living in a colony without experienced rats. Thus, six of the seven unsuccessful rats did manipulate the seesaw by sitting on the platform, but without accessing the food, i.e. ineffectual manipulation of the seesaw. The indices of concordance for (i) the identity of the rats manipulating the seesaw and (ii) if the manipulation was a success were reliably measured with values equal to 0.999 (1605/1606 observations) and 0.999 (1605/1606 observations), respectively.

### Latency to the first successful manipulation of the seesaw

**Fig. 3:**
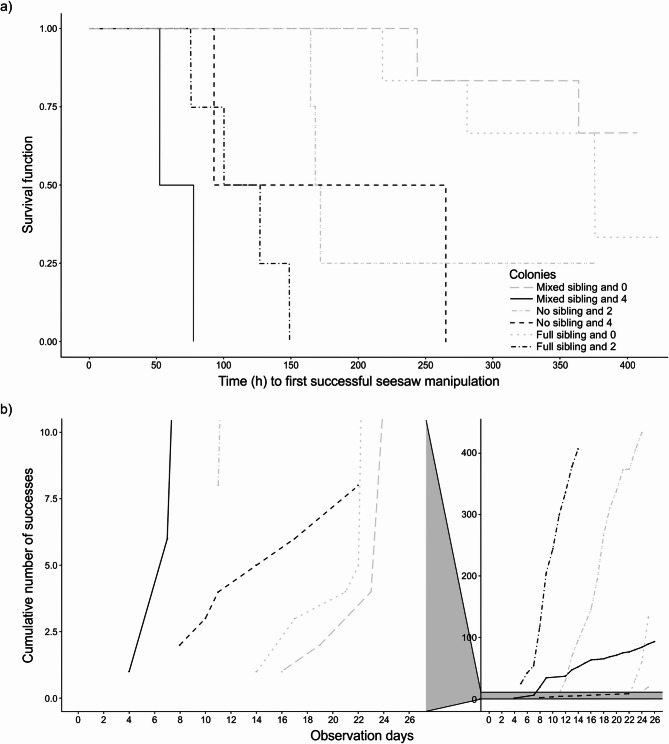
Successful manipulations of the seesaw. **(a**) Kaplan-Meier plot (“Survival function”) of the probability that individuals did not acquire the ability to successfully manipulate the seesaw until a given time in each of the colonies. Each time a naïve rat acquired the manipulation of the seesaw, the probability decreased. “Full sibling”, “Mixed sibling” and “No sibling” in the legend represent the full sibling, mixed sibling, and no sibling relatedness compositions of colonies, respectively. The numbers “4”, “2” and “0” represent the number of experienced rats living in each colony. For example, “Full sibling and 2” represents the colony with a full sibling relatedness composition and 2 trained, i.e. experienced, rats. The line types and grey scale colours for the categories are i) two dash and grey20 for “Full sibling and 2”, dotted and grey50 for “Full sibling and 0”, dashed and grey10 for “Mixed sibling and 4”, long dash and grey80 for “Mixed sibling and 0”, solid and grey0 for “No sibling and 4”, and dot dash and grey60 for “No sibling and 2”. The survival function for the colony with a full sibling relatedness composition and 0 experienced rats (dotted line) has 4 steps. The 4^th^ step is small, because the 3^rd^ and 4^th^ rats to acquire the successful manipulation of the seesaw did so after 351.53 h and 352.16 h, respectively. (**b**) The cumulative number of successes by naïve rats over the observation days for each of the 6 colonies. The left panel zooms into the data ranging from 0 to 10 cumulative number of successes, and the full data set is in the right panel.

We conducted a semi-parametric Cox proportional hazard mixed model to assess the effects of (i) the number of experienced rats in the colony, (ii) its relatedness composition, and (iii) the location of the seesaw in the enclosure as fixed effects on naïve rats’ latency to the first successful manipulation of the seesaw. We also reported full-null comparisons. The full model was a better fit to the data than the null (intercept-only) model (Χ^2^ = 14.4, *p* = 0.006). The latency to the first successful manipulation of the seesaw was shorter for naïve rats living with four experienced rats than for naïve rats living with zero experienced rats (4 vs. 0 experienced rats: *p* = 0.025, Table 1; Fig. 3a) and for naïve rats living with four experienced rats than for naïve rats living with two experienced rats (4 vs. 2 experienced rats, *p* = 0.006, Table S1, Fig. 3a). However, there was no significant difference in the latency to the first successful manipulation of the seesaw for naïve rats living with two experienced rats than for naïve rats living with zero experienced rats (2 vs. 0 experienced rats: *p* = 0.24, Table 1; Fig. 3a). These results partially supported our directional prediction for the acquisition of the successful manipulation of the seesaw as the number of experienced rats increased.

**Table 1:** Results for the rate of acquisition of the manipulation of the seesaw to access a food reward, the intermediate steps prior to the acquisition of the novel trait, the rate of performance of the manipulation of the seesaw once acquired, and innovation. “F vs M” refers to full sibling and mixed sibling relatedness compositions. “N vs M” refers to no sibling and mixed sibling relatedness compositions. The comparisons for the number of experienced rats are represented as “2 vs 0” and “4 vs 0”. “Seesaw change” refers to the change in the seesaw’s location from the initial location (I) and the location after the change (C). “HR” refers to the hazard ratio effect size. The min and max refer to the min and max values of the difference in each parameter estimate with and without each data point and represents model stability. We do not report the p value for the intercepts, because these values are meaningless. Significant results are printed in bold. A *p*-value reported with “*” represents a result that is likely a type I error.

Models	Response	Variables	Estimate (95% CI)	SE	Min	Max	HR (95% CI)	*P*
Rate to acquire a novel trait	Latency to first success	F vs. M	0.90 (−0.82–2.63)	0.88	0.48	1.09	HR: 2.47 (0.44–13.84)	0.30
		**N vs. M**	**3.88 (0.52–7.25)**	**1.72**	**2.77**	**4.53**	**HR: 48.56** **(1.68–1401.77)**	**0.024**
		2 vs. 0	−2.22 (−5.96–1.52)	1.91	−2.86	−1.17	HR: 0.11 (0.003–4.57)	0.24
		**4 vs. 0**	**2.35 (0.29–4.40)**	**1.05**	**1.72**	**2.81**	**HR: 10.44** **(1.33–81.67)**	**0.025**
		**Seesaw position: C vs. I**	**6.03 (0.95–11.11)**	**2.59**	**4.07**	**6.65**	**HR: 414.14** **(2.57–66647.90)**	**0.02**
Intermediate steps prior to acquisition of novel trait	Attending the platform	Intercept	−8.16 (−10.19 – −6.13)	1.04	−28.34	−6.56		
		F vs. M	−0.90 (−2.19–0.40)	0.66	−1.36	20.63		0.17
		N vs. M	0.22 (−0.85–1.29)	0.55	0.00	20.92		0.69
		**2 vs. 0**	**7.33 (5.17–9.49)**	**1.10**	**6.00**	**27.78**		**< 0.001**
		**4 vs. 0**	**7.61 (5.52–9.69)**	**1.06**	**6.12**	**27.89**		**< 0.001**
	Witnessing conspecifics	Intercept	−4.88 (−5.57 – −4.31)	0.31	−5.11	−4.74		
		F vs. M	0.51 (−0.31–1.26)	0.37	0.24	0.70		0.17
		N vs. M	0.21 (−0.70–1.05)	0.41	0.03	0.37		0.61
		**2 vs. 0**	**4.10 (3.41–4.99)**	**0.38**	**3.89**	**4.36**		**< 0.001**
		**4 vs. 0**	**5.07 (4.29–6.02)**	**0.41**	**4.90**	**5.25**		**< 0.001**
	Eating food rewards	Intercept	−8.18 (−11.10 – −6.56)	1.05	−28.31	−6.40		
		F vs. M	−0.98 (−2.51–0.78)	0.77	−1.55	19.55		0.21
		N vs. M	−0.12 (−1.38–1.36)	0.64	−0.46	19.82		0.85
		**2 vs. 0**	**7.84 (5.96–10.85)**	**1.14**	**6.44**	**28.34**		**< 0.001**
		**4 vs. 0**	**8.27 (6.50–11.22)**	**1.09**	**6.64**	**28.54**		**< 0.001**
Rate of performing the trait	Intervals between successes	F vs. M	1.31 (−0.29–2.87)	0.79			HR: 3.72 (0.78–17.62)	0.10
		N vs. M	1.53 (−0.17–3.24)	0.87			HR: 4.64 (0.84–25.46)	0.08
		2 vs. 0	0.10 (−1.27–1.49)	0.71			HR: 1.11 (0.28–4.44)	0.88
		4 vs. 0	−1.92 (3.51 – −0.19)	0.89			HR: 0.15 (0.03–0.83)	0.03*
		Seesaw change: C vs. I	−0.04 (−0.26–0.19)	0.12			HR: 0.96 (0.77–1.21)	0.73
Innovation	Likelihood of successful manipulations by using innovated manipulations	Intercept	−11.69 (−25.86 – −6.65)	3.20	−14.03	−10.02		
		Naïve vs. experienced	−1.66 (−17.76–58.32)	5.39	−2.69	25.58		0.76

The latency to the first successful manipulation of the seesaw by naïve rats was shorter in the no sibling relatedness composition than for the mixed sibling relatedness composition (no sibling vs. mixed sibling: *p* = 0.024, Table 1; Fig. 3a). The latency to the first successful manipulation of the seesaw by naïve rats did not differ between full sibling relatedness composition and mixed sibling relatedness composition (full sibling vs. mixed sibling: *p* = 0.30, Table 1; Fig. 3a) and between full sibling composition and no sibling relatedness composition (full sibling vs. no sibling: *p* = 0.12, Table S1, Fig. 3a). These results were not consistent with our directional relatedness composition predictions for the latency to acquire the task. The latency to the first successful manipulation of the seesaw was shorter for naïve rats after the seesaw’s location was changed than in its initial location (changed location vs. initial location: *p* = 0.02, Table 1).

The confidence intervals of some estimated parameters were quite large, which indicates an imprecision in the estimates. Nonetheless, we reported full-null model comparisons to avoid inflated type I errors, likely type I errors, and model stability values.

### Intermediate steps to the acquisition of the successful manipulation of the seesaw

**Fig. 4:**
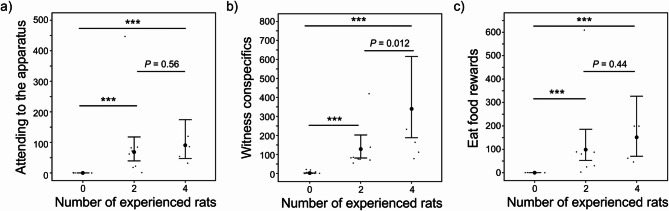
The intermediate steps prior to first success for the number of times naïve rats (**a**) attended to the apparatus, (**b**) witnessed conspecifics, and (**c**) ate food rewards when another rat lowered the platform as the number of experienced rats increased. The large black dots represent the point estimates, and the 95% CI is represented by the black whiskers. The raw data are represented by the small black dots. “***” represents *p* < 0.001.

The full models were better fits than the null (intercept-only) models (i.e. attending the platform: Χ^2^ = 26.51, *p* < 0.001; witnessing conspecifics: Χ^2^ = 27.59, *p* < 0.001; eating food rewards: Χ^2^ = 25.89, *p* < 0.001). Prior to the first successful manipulation of the seesaw, naïve rats in colonies with either 2 or 4 experienced rats (i) attended to the apparatus more often when another rat lowered the platform (4 vs. 0: *p* < 0.001; 2 vs. 0: *p* < 0.001, Table 1; Fig. 4a), (ii) witnessed conspecifics successfully manipulate the seesaw more often (4 vs. 0: *p* < 0.001; 2 vs. 0: *p* < 0.001, Table 1; Fig. 4b), and (iii) ate more often when another rat lowered the platform (4 vs. 0: *p* < 0.001; 2 vs. 0: *p* < 0.001, Table 1; Fig. 4c) than naïve rats in colonies with 0 experienced rats. Naïve rats in colonies with 4 experienced rats witnessed conspecifics successfully manipulate the seesaw more often than naïve rats in colonies with 2 experienced rats (4 vs. 2: Estimate ± SE = 0.97 ± 0.39, *p* = 0.012; Fig. 4b, Table S1). There were no differences between naïve rats in colonies with 4 or 2 experienced rats in how often they (i) attended to the apparatus prior to the first successful manipulation when another rat lowered the platform (4 vs. 2: Estimate ± SE = 0.28 ± 0.48, *p* = 0.56; Fig. 4a, Table S1), and (ii) ate prior to the first successful manipulation when another rat lowered the platform (4 vs. 2: Estimate ± SE = 0.43 ± 0.56, *p* = 0.44; Fig. 4c, Table S1).

Naïve rats in colonies with a full sibling relatedness composition attended to the apparatus less often than naïve rats in colonies with a no sibling relatedness composition (full sibling vs. no sibling: Estimate ± SE = −1.12 ± 0.40, *p* = 0.005, Table S1). Otherwise, the relatedness compositions of colonies did not affect how often naïve rats (i) attended to the apparatus prior to the first successful manipulation when another rat lowered the platform (full sibling vs. mixed sibling: *p* = 0.17; no sibling vs. mixed sibling: *p* = 0.69, Table 1), (ii) witnessed conspecifics successfully manipulate the seesaw (full sibling vs. mixed sibling: *p* = 0.17; no sibling vs. mixed sibling: *p* = 0.61, Table 1; full sibling vs. no sibling: Estimate ± SE = 0.30 ± 0.34, *p* = 0.38, Table S1), and (iii) ate prior to the first successful manipulation when another rat lowered the platform (full sibling vs. mixed sibling: *p* = 0.21; no sibling vs. mixed sibling: *p* = 0.85, Table 1; full sibling vs. no sibling: Estimate ± SE = −0.86 ± 0.46, *p* = 0.064, Tables S1). These results were not consistent with our directional prediction for the effect of the relatedness composition on the intermediate steps to the acquisition of the successful manipulation of the seesaw.

### Rate of seesaw performance once acquired

The full-null model comparison showed that the full model was not a better fit than the null (intercept-only) model (Χ^2^ = 8.63, *p* = 0.07), so any significant effects are likely type I errors^23^. Intervals between successful manipulations of the seesaw were shorter for naïve rats living with four experienced rats than for naïve rats living with zero experienced rats (4 vs. 0: *p* = 0.03, Table 1; Fig. 3b, however this is likely a type I error) and for naïve rats living with four experienced rats than for naïve rats living with two experienced rats (4 vs. 2: Estimate ± SE = −2.03 ± 0.83, *p* = 0.01, however this is likely a type I error, Table S1). Intervals between successful manipulations of the seesaw did not differ between naïve rats living with two experienced rats compared to naïve rats living with zero experienced rats (2 vs. 0: *p* = 0.88, Table 1; Fig. 3b). In other words, the number of experienced rats did not clearly affect the rate of seesaw performance once acquired. Intervals between successful manipulations of the seesaw did not differ between relatedness compositions (full sibling vs. mixed sibling: *p* = 0.10; no sibling vs. mixed sibling: *p* = 0.08, Table 1; full sibling vs. no sibling: Estimate ± SE = −0.22 ± 0.78, *p* = 0.78, Table S1). The change in the seesaw’s location did not affect the intervals between successful seesaw manipulations (changed location vs. initial location: *p* = 0.73, Table 1).

### Innovations

**Fig. 5:**
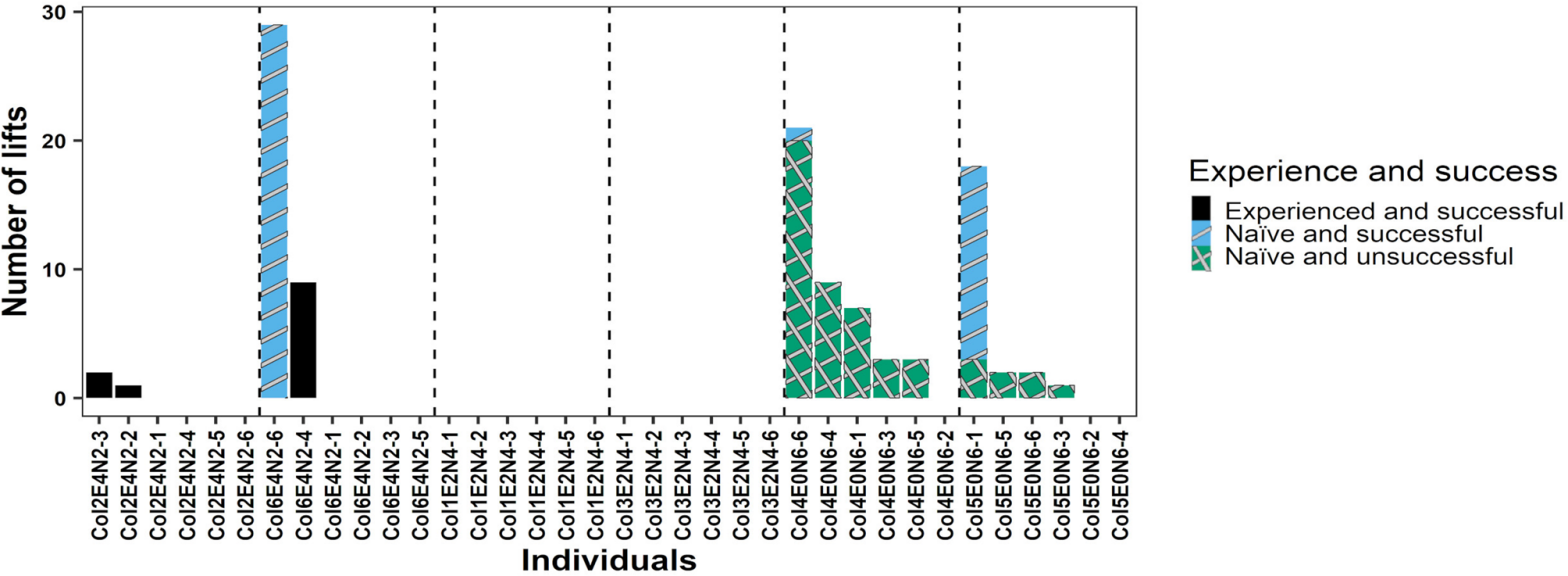
The cumulative number of successful and unsuccessful manipulations of the seesaw by directly lifting the lid to access food (i.e. without operating the seesaw mechanism by sitting on the platform) for experienced and naïve rats in the six colonies. The figure is ordered by the decreasing number of experienced rats by enclosure, by the decreasing relatedness composition, and by the decreasing number of lifts of the lid. In two enclosures with experienced rats, individuals innovated food acquisition by lifting the lid directly: in enclosure 2, one experienced rat innovated lifting the lid to manipulate the seesaw and the other experienced rat subsequently learned this modified behaviour; in enclosure 6, one experienced rat and one naïve rat innovated lifting the lid directly to get access to food. For naïve rats in enclosures without experienced rats, only 1 rat in both enclosures innovated food acquisition successfully by lifting the lid directly. All other naïve rats in these enclosures failed if trying at all. Individual identities on the abscissa are denoted like in Fig. 2. The legend’s “Experience and success” refers to experienced and naïve rats and successful and unsuccessful manipulations. The bars for the categories are stacked i) black for “Experienced and successful”, ii) light blue with a stripe for “Naïve and successful”, and ii) green with a crosshatch for “Naïve and unsuccessful”. The colours are from a colour-blind palette.

Rats exhibited a novel or modified behaviour to operate the seesaw in 4 of the 6 colonies (see Supplementary Information, S3). Naïve rats living in colonies with 0 experienced rats innovated behaviours to successfully manipulate the seesaw by sitting on the platform (Figs. 2 and 3) or by directly lifting the lid without operating the seesaw mechanism by sitting on the platform (Fig. 5). Naïve and experienced rats in two other colonies (colonies 2 and 6, both having 2 experienced rats and 4 naïve rats) successfully manipulated the seesaw by directly lifting the lid to successfully obtain the food reward, which no experienced rat had shown before (Fig. 5). Thirteen out of 36 rats in total (36.1%) attempted to directly lift the lid to manipulate the seesaw rather than sit on the platform to manipulate the seesaw. Only 6 rats successfully manipulated the seesaw by directly lifting the lid. Lifting the lid directly without sitting on the platform to manipulate the seesaw represented less than 1% of successful manipulations of the seesaw in our study (57 out of 5909 successful manipulations, i.e. 0.96%). The mean number of lifting attempts per rat, excluding rats that did not attempt to lift, was 5.36 ± 2.72. The full-null model comparison showed that the full model was not a better fit than the null (intercept-only) model (Χ^2^ = 4.82, *p* = 0.09). The likelihood of successful manipulations of the seesaw by using innovated manipulations (i.e. directly lifting the lid or sitting on the platform for the rats living in colonies without experienced rats; directly lifting the lid for rats living in colonies with experienced rats) did not differ between naïve and experienced rats (naïve rats vs. experienced rats: *p* = 0.76, Table 1).

## Discussion

Our results provide evidence consistent with social transmission affecting the rate of acquisition, but not the rate of performance, of a non-intuitive food provisioning task by social learning in wild-type Norway rats living in semi-natural colonies. The results partially supported our directional prediction that the latency to the first successful manipulation of the seesaw should decrease as the number of experienced rats in the colony increases. Naïve rats living without experienced rats apparently first needed asocial learners to acquire the trait by trial-and-error learning while interacting with the physical environment, which was followed by social transmission within their colonies. Our results are consistent with previous laboratory studies that reported a decrease in the latency to acquire food preferences in Norway rats^7^, feeding behaviour in pigeons, *Columba livia*^6^, and food site preference in guppies, *Poecilia reticulata*^8^ as the number of demonstrators present increased. In previous studies, naïve individuals were not living and interacting with experimentally manipulated numbers of demonstrators throughout the study.

Individuals who socially learn are information scroungers, whereas individuals who learn asocially are information producers^24,25^. Asocial learners may incur greater temporal and energetic costs from interacting with the environment compared to social learners, for whom information acquisition from observing others seems cheap^26^. Naïve rats living with 2 and 4 experienced rats had access to additional social information, i.e. attending to the apparatus, witnessing conspecifics successfully manipulating the seesaw and eating food rewards, prior to the first successful manipulation of the seesaw than naïve rats living with 0 experienced rats, which can explain the shorter latencies to first successful manipulation of the seesaw for naïve rats living with either 2 or 4 experienced rats than for naïve rats living without experienced rats. Naïve rats living with 4 experienced rats witnessed conspecifics successfully manipulating the seesaw more often than naïve rats living with 2 experienced rats. Experienced rats living in the two colonies with 4 experienced rats successfully manipulated the seesaw 1814 and 1772 times, respectively, whereas experienced rats living in the two colonies with 2 experienced rats successfully manipulated the seesaw 551 and 678 times (Fig. 2). Thus, the naïve rats living with 4 experienced rats had more opportunities to observe the experienced rats successfully manipulating the seesaw than the naïve rats living with 2 experienced rats, which likely explains why the latency to the first successful manipulation of the seesaw was shorter for naïve rats living with 4 than with 2 experienced rats. Consistent with these arguments, in previous laboratory studies observing a greater number of demonstrators, without interacting with the demonstrators, decreased the latency to acquire traits for naïve individuals^6–8^, and the rate of acquisition was lower for naive subjects without demonstrators than for subjects with demonstrators^27–29^.

Previous studies found that the social transmission of food preferences in wild-type Norway rats housed under laboratory conditions were independent of relatedness and familiarity^15,16^, and unrelated wild-type males reciprocated received food provisioning more often than full brothers^30^. In a controlled laboratory experiment, wild-type female Norway rats exchanged food with social partners by applying the direct reciprocity decision rule, which states help someone who previously helped you, rather than copying by imitation^31^. Consistent with previous findings of social learning in Norway rats^15,16^, our directional predictions for the effect of relatedness composition of colonies on the social learning propensity of experimental subjects were not supported.

We found evidence for innovations in the form of a new or modified behaviour not previously observed in four of six colonies. Individuals can acquire information about the environment by using trial-and-error learning while interacting with the environment, i.e. making use of personal information^21^. Innovators introduced lifting the lid directly to manipulate the seesaw without sitting on the platform, which started to spread within colonies before the end of the study. Lifting the lid directly might have spread to more colony members if the rats had invented this practice earlier so that their colony mates would have had more time to acquire social information to learn this alternative method of obtaining the food. This suggestion is partially supported by the observation that 15 more rats were lifting the lid directly to access food 1 month later during training sessions for a subsequent study of cooperation (unpublished data).

A limitation of this study is that the confidence intervals for some estimated parameters were quite large, which reflects an imprecision in the estimates. However, we reported full-null model comparisons to avoid inflated type I errors, likely type I errors, and model stability values. The sample size for naïve Norway rats in our study was 24 individuals, distributed over six colonies. Replication of this study with a larger sample size would be warranted in the future.

We did not design this study with the goal to specify the precise social learning mechanisms of Norway rats that acquired and performed the trait, after acquiring it, as such we cannot distinguish between different social learning processes. Thorpe^32^ proposed that the simplest social learning processes, such as local enhancement, were operating in wild populations. In our study, the latency to the first successful manipulation of the seesaw increased after the location of the seesaw was changed, which may be more likely explained by more rats acquiring the trait later in the study than before the change in location than by local enhancement. In previous studies, excretory markings of demonstrators affected the social transmission of food preferences in Norway rats^33,34^. Our experienced rats lived with the naïve rats for nearly 1 month, and potential excretory markings of experienced rats were not removed following each manipulation of the seesaw by an experienced rat. Thus, stimulus enhancement is one possible social learning mechanism that may have affected our results. A series of experiments would be needed to test for the operating social learning mechanisms, e.g. local enhancement and stimulus enhancement, which could be more easily done using experimental laboratory techniques than studying semi-natural colonies^2^.

This study supported the social learning hypothesis and showed that social information for a non-intuitive food provisioning task can be socially transmitted through semi-natural colonies of wild-type Norway rats. Naïve rats successfully manipulated the seesaws sooner when living with four experienced rats compared to living (i) with two experienced rats or (ii) without experienced rats. The latency to successfully manipulate the seesaw did not decrease as the relatedness composition increased, however naïve rats living without siblings successfully manipulated the seesaw sooner than naïve rats living with a mix of siblings and non-siblings. The number of times naïve rats were near the seesaw when another rat manipulated the seesaw, i.e. witness conspecifics manipulating the seesaw, and ate food rewards when another rat manipulated the seesaw prior to own trait acquisition by naïve rats were intermediate steps to social learning supporting that naïve rats acquired information from conspecific prior to acquiring the trait. The intervals between successful manipulations were not affected by the number of experienced rats and the relatedness composition of colonies.

## Methods

### Model system

Knowledge of the behaviours of wild Norway rats is rather limited^35–37^. Colony size can reach over 150 individuals^36^. Natural colonies are structured in variable subgroups ranging from single individuals of either sex to pairs, unisex groups and harems with and without offspring^35,37^. Norway rats caught at 9 sites had little genetic relatedness among individuals caught at the same sites, but showed high levels of genetic diversity and genetic structuring across small geographic distances^38^.

Field studies of Norway rats, *Rattus norvegicus*, anecdotally reported what appeared to be social transmission of foraging behaviours and food preferences^35,39^, which was subsequently supported by laboratory studies done with inbreed lab strains of Norway rats^27,40,41^ and wild-type Norway rats^42^. Young Norway rats learned from conspecifics where, when and what to eat^42^, whereas adult male Norway rats learned food preferences from excretory markings and gustatory cues of conspecifics^33,34^. Furthermore, adult male Norway rats improved their foraging efficiency in the presence of trained demonstrators with food available, and they showed a shorter latency to start digging and a greater number of food items dug up in this condition^27^.

Norway rats are highly social animals^43,45^ that can distinguish between kin and non-kin^44,45^, between different degrees of relatedness^46^, between colony members and intruders^47^ and between single individuals (i.e. true individual recognition^48^. We used Norway rats that are well-known for their capacity to cooperate by using detailed information from their social partners^30,49–54^. Norway rats account for their partners’ need^50,51,55^, solicitation^56–58^ and helpfulness^52,54,59–61^ when cooperating. These and other studies illustrate the capacity of Norway rats to respond appropriately to social cues^62^ Most studies of Norway rats have been done in the laboratory. To study the behaviour of rats under semi-natural conditions, we established six colonies of wild-type Norway rats in outdoor enclosures.

### Experimental subjects and housing conditions

Fifty-six outbred, female wild-type Norway rats, *Rattus norvegicus* (source: Behavioural Physiology Unit, Groningen Institute of Evolutionary Life Sciences, University of Groningen, The Netherlands) were brought to the Ethologische Station Hasli of the University of Bern, Switzerland. The rats were individually marked by a white hair dye (rats were habituated to the smell and application) and by ear punches. The patterns of the hair dye allowed us to identify each individual rat within a colony. If blood was visible after ear punching, we stopped it by gently pressing on the ear with a paper tissue for 10 s. The rats were housed indoors in groups of 4 to 6 littermates per housing cage (80 cm x 50 cm x 37.5 cm). To avoid male-male competition for females and possibly deaths caused by overt aggression, only females were included in the formation of each colony. The rats were habituated to handling (see Supplementary Information, S2 for more information). The study is reported in accordance with the ARRIVE guidelines. The license to perform animal experiments was provided by the Swiss Federal Veterinary Office of the Canton of Bern (license number BE 55/18) to M.T. The ticket for indispensable research was provided by the University of Bern (ticket number EAC-201216-T#212) to M.T. The experiment was performed in accordance with relevant guidelines and regulations.

Following 6 weeks of acclimatisation, i.e. gradual temperature decrease, the rats moved into outdoor enclosures under semi-natural environmental conditions. Each enclosure (294 cm x 208 cm x 258 cm) consisted of (i) a cement floor covered with 5 cm of soil, and stainless-steel walls, (ii) an area of soil (132 cm x 105 cm, and 40 cm deep) for digging and building tunnels, (iii) 3 wooden shelters, (iv) 3 heat lamps (turned on when the temperature was < 6˚C), (v) 2 PVC tubes, (vi) 2 pieces of wood, (vii) 4 infrared light bulbs (Supplementary Information, Fig. S1), and (viii) a Raspberry Pi Model 3 B + with a Raspberry Pi camera H with a fisheye lens and night vision to record videos with a frame rate of 30 frames/s and a resolution of 1024 frame width by 768 frame height. Hay and straw were provided weekly for the rats to build nests. Grain mix was additionally provided five times a week, and fresh fruits or vegetables were provided twice a week. We performed daily, weekly and monthly health checks.

### Apparatus

We provided 1 seesaw per colony. Each seesaw consisted of a platform connected by a lever to a lid, which covered food rewards in a food box. The seesaw rested on a PVC base (102 cm x 72 cm x 0.5 cm, Fig. 1a and c). Rats could lower the platform by sitting on it, but they could also access the food by lifting the covering lid (Fig. 1b and d). To dissuade rats from accessing the food rewards by lifting the lid, it was surrounded by 4 pieces of PVC, which made lid-lifting difficult. A similar seesaw was used to study cooperation in keas, *Nestor notabilis*^22^. When a rat lowered the platform, this was connected with an electromagnet and 2 microswitches (Fig. 1d). The electromagnet connected with a microswitch, a 24 V power supply, and a time relay, which kept the food tray open for 63 s. The second microswitch connected with a Raspberry Pi Model 3 B+, which automatically logged the dates and times when the seesaw was opened and closed. Data were relayed from the Raspberry Pi to a server via an Ethernet cable.

### Main study: training

In the pilot study, we used 20 rats (see Supplementary Information, S3), and in the main study we tested 36 rats. To experimentally induce seesaw manipulations, 12 rats were randomly selected from different families for training. Each rat was placed in an experimental cage (80 cm x 50 cm x 37.5 cm) with the seesaw in the training room and returned to its housing cage after each training session. A successful manipulation of the seesaw was defined as lowering the platform by sitting on the platform or by lifting the lid and eating the food reward. An unsuccessful manipulation of the seesaw was defined as: 1) not lowering the platform all the way down to connect with the microswitches and the electromagnet, or ii) lowering the platform without eating the food reward. The food rewards were peanut halves for the first 5 successful sessions to increase the motivation to manipulate the seesaw, and were then changed to oats. An observer recorded the number of successful and unsuccessful manipulations. The criterion to consider a rat as trained, hereafter ‘experienced rats’, was ≥ 4 successful manipulations per 15 min session on 2 consecutive days (the criterion was met after 19 to 26 training sessions). Trained rats successfully manipulated the seesaw by sitting on the platform and not by attempting to lift the lid.

### Main study: procedure

We experimentally introduced seesaw manipulations in 4 of the 6 colonies and manipulated the number of experienced rats and the relatedness composition of each colony (6 rats/colony). The rats had 1 week to habituate to the new physical and social environment, and the mean mass and age of rats were 193 g ± 5 g and 72 days ± 0.5 days, respectively. There were 4 experienced rats in 2 colonies, 2 experienced rats in 2 colonies, and 0 experienced rats in 2 colonies (Table 2). There were full siblings (all sisters) in 2 colonies, no siblings (no sisters) in 2 colonies, and mixed siblings (3 pairs of 2 sisters) in 2 colonies (Table 2). Naïve rats had no previous experience with the seesaw. The study lasted 26 observation days from November 28th, 2019 to December 31 st, 2019 and yielded 2,195.42 h of video recordings. The study ran under red light conditions at night, with daily temperatures between 0 °C and 8 °C.

**Table 2:** Colony compositions. Each colony contained 6 rats. Number of experienced rats represents rats trained to open the seesaw by sitting on the platform.

Colony No.	Relatedness composition	Number of siblings	Number of experienced rats	Number of naïve rats
1	Full Sibling	6	2	4
2	Mixed Sibling	3 * 2 sisters	4	2
3	No Sibling	0	2	4
4	Full Sibling	6	0	6
5	Mixed Sibling	3 * 2 sisters	0	6
6	No Sibling	0	4	2

The seesaw was covered by a cage top each morning to prevent access, and it was uncovered in the evening so that the rats had access to it overnight during their active period. The food box was filled with oats as food rewards. To account for local enhancement, the position of the seesaw was changed by moving the seesaw to a different location inside the enclosures after the first 15 days (Supplementary Information, Fig. S1a and Fig. S1b). Researchers looked at the videos and recorded the identity of the rats that (i) lowered the platform, (ii) witnessed conspecifics successfully manipulate the seesaw from any location in the enclosure, except when in the food cage, houses or the area with soil (Fig. S1), (iii) attended to the apparatus, i.e. were present on the base of the apparatus when it was manipulated by a conspecific as a proxy of witnessing conspecifics manipulate the seesaw, (iv) ate the food reward, and (v) manipulated the seesaw in a new or modified way (innovation), such as lifting the lid rather than sitting on the platform. For each naïve rat, the latency to the first successful manipulation of the seesaw was recorded as the time from the start of the experiment in the colony to the first successful manipulation of the seesaw. If a naïve rat did not successfully manipulate the seesaw at all during the study, this latency was determined as the time from the start of the experiment in the colony to the end of the study, and we recorded that the event did not occur. For each naïve rat that subsequently acquired the successful manipulation of the seesaw, we recorded the intervals between successful manipulations of the seesaw, starting from the interval between the 1 st and 2nd successes, then between the 2nd and 3rd successes and so forth, until the end of the study. The number of successful and unsuccessful manipulations of the seesaw were recorded.

### Statistical analysis

To assess the inter-observer reliability, we calculated the index of concordance between the 2 observers for the identity of rats manipulating the seesaw and if the manipulation was a success^63^. To investigate for effects on naïve rats’ latencies to the first successful manipulation of the seesaw, i.e. the acquisition of a novel behavioural trait, we ran a semi-parametric Cox proportional hazard mixed model to assess the effects of (i) the number of experienced rats in the colony, (ii) its relatedness composition, and (iii) the location of the seesaw in the enclosure as a time dependent covariate as fixed effects on naïve rats’ latency to the first successful manipulation of the seesaw. Colony number was included as a random intercept effect, and there were no theoretically important random slopes. We compared the full model to the null model, i.e. without the number of experienced rats and the relatedness composition. The proportional hazard assumption was met^64^.

The effects of the number of experienced rats and relatedness composition of colonies on the latency to first successful manipulation of the seesaw may be explained by the occurrence of potential intermediate steps in the acquisition of social information prior to the first successful manipulation of the seesaw. We assessed if intermediate steps influenced the acquisition of the first successful manipulation of the seesaw, and each model is a proxy of naïve rats witnessing conspecifics manipulating the seesaw A generalized linear mixed model with a quasi-Poisson (Poisson lognormal) distribution was conducted to assess the influence of the number of experienced rats and the relatedness composition of colonies on the number of times each naïve rat attended to the apparatus when another rat lowered the platform, prior to first success. A generalized linear mixed model with a quasi-Poisson (Poisson lognormal) distribution was conducted to assess the influence of the number of experienced rats and the relatedness composition of colonies on the cumulative number of times each naïve rat witnessed another rat successfully manipulating the seesaw, prior to first success. A generalized linear mixed model with a quasi-Poisson (Poisson lognormal) distribution was conducted to assess the influence of the number of experienced rats and the relatedness composition of colonies on the cumulative number of times each naïve rat ate the reward when another rat lowered the platform, prior to first success. For these models, colonies and an observation-level value^65^ were the random intercept effects, and we applied an offset to account for each rat’s latency to the first successful manipulation of the seesaw or till the end of the study for rats that did not acquire the task. We accounted for all theoretically important random slopes. We compared the full models to the null models.

To test for effects on the intervals between successful manipulations of the seesaw, i.e. the rate of performance of the acquired trait, we ran a parametric event history analysis with a Weibull distribution, since the proportional hazard assumption was not met. The fixed effects were (i) the number of experienced rats in the colony, (ii) its relatedness composition, and (iii) the location of the seesaw in the enclosure as a time dependent covariate, and we included rat identities and colonies as random effects, i.e. shared gamma frailty. We compared the full model to the null model, i.e. without the number of experienced rats and the relatedness composition.

A linear mixed model with a Gaussian distribution was conducted to assess the influence of the experience of the previous rat (naïve vs. experienced) and the kinship of the rats (sister vs. non-sister), as fixed effects, on the latency for naïve rats to successfully manipulate the seesaw after it was manipulated by another rat. The colonies and individual rats were random intercept effects. The model residuals were normally distributed when we log-transformed the response variable, i.e. the latency for naïve rats to successfully manipulate the seesaw after it was manipulated by another rat. We accounted for all theoretically important random slopes. We compared the full model to the null model.

We recorded the identity of rats that innovated, i.e. developed new or modified behaviour to successfully manipulate the seesaw, and we recorded how often individual rats successfully and unsuccessfully manipulated the seesaw by using these new or modified techniques. Lifting the lid rather than sitting on the platform is an alternative way to successfully manipulate the seesaw, which none of the experienced rats learned during the training sessions. A generalized linear mixed model with a binomial distribution was used to assess the experience of rats, i.e. naïve or experienced, on the likelihood of successful manipulations of the seesaw by using innovated manipulations (i.e. lifting the lid and sitting on the platform for the rats living in colonies without experienced rats; lifting the lid for rats living in colonies with experienced rats). The rat identities and the colonies were random intercept effects. There were few successful openings of the seesaw by using innovations, therefore we only included the experience of rats, i.e. naïve or experienced, as a fixed effect. We compared the full model to the null model.

To test the significance of the fixed effects of interest, we ran full-null model comparisons to avoid cryptic multiple testing, avoiding multiple testing and highly inflated type I errors^23^. We ran the same models with different levels of comparison (e.g. 4 vs. 2 experienced rats and full sibling vs. no sibling). This does not increase the type I error rate and it is not multiple testing, since we were not running a different model with a different set of variables. We assessed the model stability based on DFBetas and reported the minimum and maximum values, which represent the minimum and maximum values of the difference in each parameter estimate with and without each data point. We used R version 4.0.3^66^ with the frailtyPenal function of the “frailtypack”^67,68^, “survival”^69,70^, “coxme”^71^, “survminer”^72^, “lme4”^73^, “ggplot2”^74^ and “multcomp”^75^ packages. Throughout the paper, means and estimated coefficients are reported with their standard error, unless otherwise stated; an alpha of 0.05 was adopted.

## Supplementary Information

Below is the link to the electronic supplementary material.

Supplementary Material 1

Supplementary Material 2

Supplementary Material 3

Supplementary Material 4

Supplementary Material 5

Supplementary Material 6

Supplementary Material 7

Supplementary Material 8

Supplementary Material 9

Supplementary Material 10

## Data Availability

We have uploaded the codes (.r) and data files (.RData) to the submission. If accepted, they will be added to a public repository or as part of the Supplementary Information. The codes can be opened in R or Rstudio. All the data files are.RData files, which can be opened in R or Rstudio. The codes to open the data files are in the codes file, e.g. load(“latency_first_success_naive_rats.RData”). If you have problems opening the data files, please send SCE an email.
