# Supplementary material for: Experimental evidence for social learning in semi-natural, wild-type Norway rats
Source: Sci Rep. 2025 Oct 27;15:37364. doi: 10.1038/s41598-025-25316-6 (PMC12559377; doi:10.1038/s41598-025-25316-6)
Supplement: Supplementary file 8 — Supplementary Material 8 [file 41598_2025_25316_MOESM8_ESM.docx]

**Supplementary information**

**Article title: Experimental evidence for social learning in semi-natural, wild-type Norway rats**

**Authors: Sacha C. Engelhardt, Harshkumar Vasoya, and Michael Taborsky**

**Email of corresponding author:** [sacha.engelhardt@uni-goettingen.de](mailto:sacha.engelhardt@uni-goettingen.de)

**S1 Additional statistical analyses**

**Table S1 Additional model results for the rate of acquisition of the manipulation of the seesaw to access a food reward, intermediate steps prior to the acquisition of the novel trait, and the rate of performance of the manipulation of the seesaw once acquired.** For these additional models, we changed the reference levels for the relatedness composition and the number of experienced rats to “no sibling” and “2”, respectively. These additional models do not increase the type I error rate and is not multiple testing, since we are not running a different model with a different set of variables. “M vs N” refers to mixed sibling and no sibling relatedness compositions, respectively. “F vs N” refers to full sibling and no sibling relatedness compositions, respectively. The comparisons for the number of experienced rats are represented as “0 vs 2” and “4 vs 2”. “Seesaw change” refers to the change in the seesaw’s location from the initial location (I) and the location after the change (C). “HR” refers to the hazard ratio effect size. The min and max refer to the min and max values of the difference in each parameter estimate with and without each data point and represents model stability. We do not report the p value for the intercepts, because these values are meaningless. Significant results are printed in bold. A *p*-value reported with “*” represents a result that is likely a type I error (see main text for explanation).

| Models | Response | Variables | Estimate (95% CI) | SE | Min | Max | HR (95% CI) | P |
| --- | --- | --- | --- | --- | --- | --- | --- | --- |
| Rate to acquire a novel trait | Latency to first success | **M vs N** | **-3.88 (-7.25 – -0.52)** | **1.72** | **-4.53** | **-2.77** | **HR: 0.02**  **(0.001 – 0.59)** | **0.02** |
|  |  | F vs N | -2.98 (-6.71 – 0.76) | 1.90 | -3.57 | -1.90 | HR: 0.05  (0.001 – 2.13) | 0.12 |
|  |  | 0 vs 2 | 2.22 (-1.52 – 5.96) | 1.91 | 1.17 | 2.86 | HR: 9.22  (0.22 – 388.32) | 0.24 |
|  |  | **4 vs 2** | **4.57 (1.32 – 7.82)** | **1.66** | **3.92** | **4.89** | **HR: 96.27**  **(3.74 – 2478.79)** | **0.006** |
|  |  | **Seesaw position: C vs I** | **6.03 (0.95 – 11.11)** | **2.59** | **4.07** | **6.65** | **HR: 414.14**  **(2.57 – 66647.90)** | **0.02** |
| Intermediate steps prior to acquisition of novel trait | Attending the platform | Intercept | -0.61 (-1.14 – -0.07) | 0.27 | -21.43 | -0.49 |  |  |
|  |  | M vs N | -0.22 (-1.29 – 0.85) | 0.55 | -127.05 | 0.00 |  | 0.69 |
|  |  | **F vs N** | **1.12 (-1.90 – 0.34)** | **0.40** | **-1.52** | **20.64** |  | **0.005** |
|  |  | **0 vs 2** | **-7.33 (-9.49 – -5.17)** | **1.10** | **-27.81** | **-6.00** |  | **< 0.001** |
|  |  | 4 vs 2 | 0.28 (-0.65 – 1.21) | 0.48 | 0.11 | 20.99 |  | 0.56 |
|  | Witnessing conspecifics | Intercept | -0.57 (-1.13 – 0.002) | 0.26 | -0.77 | -0.49 |  |  |
|  |  | M vs N | -0.21 (-1.05 – 0.70) | 0.41 | -0.37 | -0.03 |  | 0.61 |
|  |  | F vs N | 0.30 (-0.46 – 1.03) | 0.34 | 0.21 | 0.48 |  | 0.38 |
|  |  | **0 vs 2** | **-4.10 (-4.99 – -3.41)** | **0.38** | **-4.36** | **-3.89** |  | **< 0.001** |
|  |  | **4 vs 2** | **0.97 (0.11 – 1.79)** | **0.39** | **0.84** | **1.13** |  | **0.01** |
|  | Eating food rewards | Intercept | -0.46 (-1.22 – 0.21) | 0.32 | -20.48 | -0.38 |  |  |
|  |  | M vs N | 0.12 (-1.36 – 1.38) | 0.64 | -31.87 | 0.46 |  | 0.85 |
|  |  | F vs N | -0.86 (-1.83 – 0.22) | 0.46 | -1.33 | 20.11 |  | 0.06 |
|  |  | **0 vs 2** | **-7.84 (-10.85 – -5.96)** | **1.14** | **-35.34** | **-6.44** |  | **< 0.001** |
|  |  | 4 vs 2 | 0.43 (-0.69 – 1.71) | 0.56 | 0.19 | 20.32 |  | 0.44 |
| Rate of performing the trait | Intervals between successes | M vs N | -1.53 (-3.22 – 0.22) | 0.89 |  |  | HR: 0.22  (0.04 – 1.25) | 0.09 |
|  |  | F vs N | -0.22 (-1.77 – 1.30) | 0.78 |  |  | HR: 0.80  (0.17 – 3.68) | 0.78 |
|  |  | 0 vs 2 | -0.10 (-1.51 – 1.29) | 0.71 |  |  | HR: 0.90  (0.22 – 3.62) | 0.88 |
|  |  | 4 vs 2 | -2.03 (-3.51 – -0.40) | 0.83 |  |  | HR: 0.13  (0.03 – 0.67) | 0.01* |
|  |  | Seesaw change: C vs I | -0.04 (-0.26 – 0.19) | 0.12 |  |  | HR: 0.96  (0.77 – 1.21) | 0.73 |

***S2 Additional housing conditions***

Housing cages measured 80 cm x 50 cm x 37.5 cm and consisted of litter, a wooden shelter, a tunnel, paper toys and a wooden block. The rats were habituated to handling by the experimenters within days of their arrival. The rats were kept in a room with temperature and humidity close to natural temperature and humidity. From weaning till the end of the study, the food regime was kept the same. Conventional rat pellets and water were provided daily ad libitum. Grain mix was additionally provided three times a week, and fresh fruits/vegetables were provided twice a week. Food and water were provided in an open cage, which measured 80 cm x 50 cm x 37.5 cm. Rats were handled regularly to keep them habituated to the experimenters. To uniquely identify rats, they were marked with unique hair dyes (organic bleach Oway Hbleach and HCatalyst), which the rats were habituated to the smell and application once per week prior to the study. The light regime followed the natural light/dark cycle. As rats are primarily nocturnal (Barnett, 1963) and their pigment in the eye has a λ max value of 510 nm (Yokoyama & Radlwimmer, 1998), all stages of the experiment were conducted under red light (625–740 nm) conditions during the night. The 36 main study rats were part for additional behavioural studies.


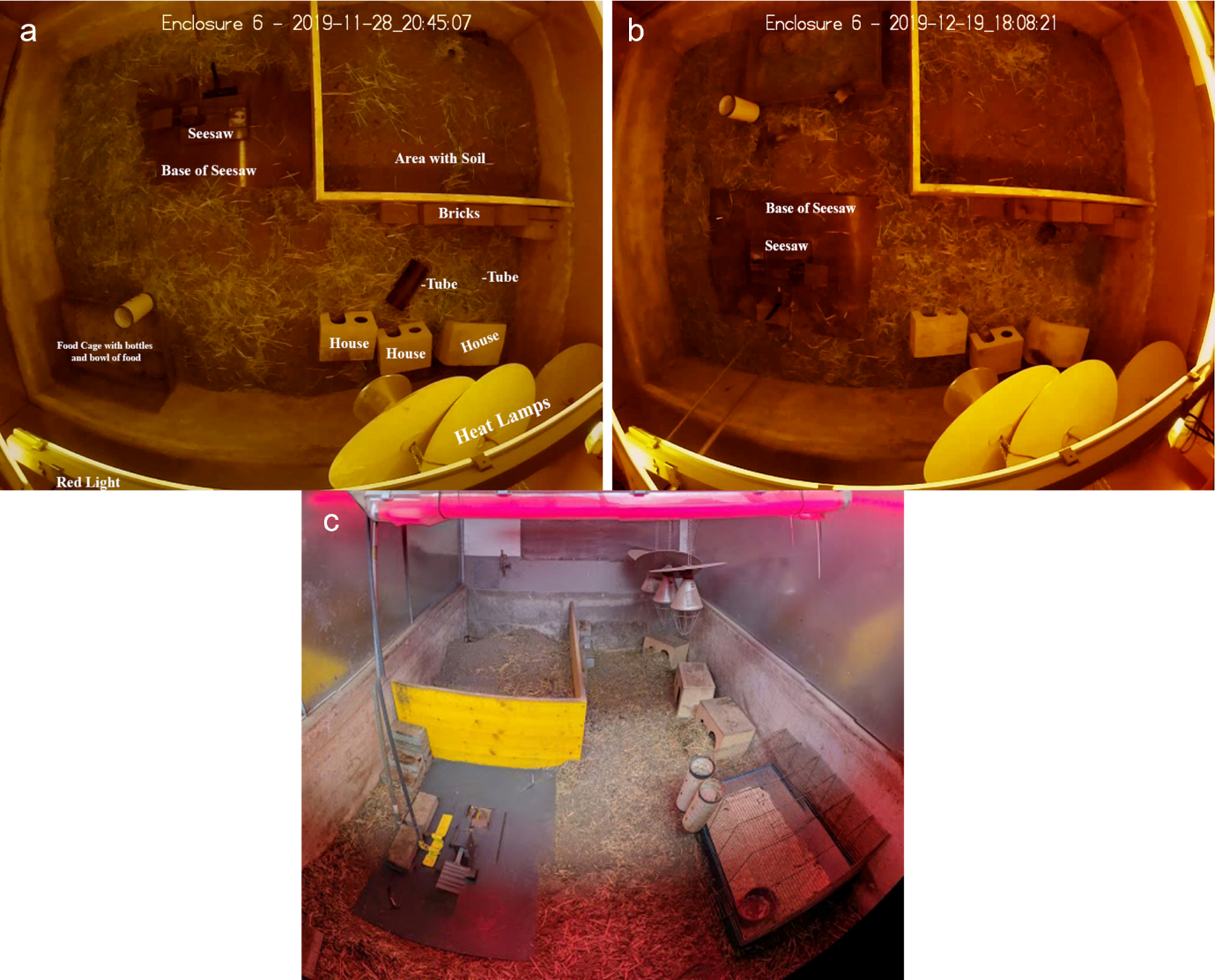


**Fig. S1** The set-up in each enclosure. a) An enclosure and the seesaw, as seen from the Raspberry pi camera at night, and the houses, the heat lamps, the food cage, and the area with soil are visible. b) The seesaw position and the food cage were moved to account for local enhancement. c) An enclosure with all the materials and the seesaw in the morning, during daylight

***S3 Pilot study***

We described the experimental subjects and housing conditions in the main text (see the subsection “Experimental Subjects and Housing Conditions” of the Methods). We performed a pilot study with 20 females. We randomly selected which individuals would be combined to form colonies, and we randomly selected which of these individuals would be trained with a seesaw to experimentally induce innovations. Four rats were selected from 2 families of sisters for training, and each rat received 14 solo training sessions, which each lasted 15 minutes. Training sessions were performed in a separate room from the housing room. Training sessions occurred in a cage with the dimensions of 80 cm x 50 cm x 37.5 cm. The food rewards for operating the seesaw were peanut halves for the first 5 sessions to increase the motivation of the rats, and the food reward was subsequently changed to oats. We randomized the order of rats for each training session. The observer was in the room during training sessions. A successful manipulation of the seesaw was defined as a rat opening the food box by lowering the platform, which raised the lid on top of the food box, and eating the food reward from the food box. Rats were considered as trained when they could successfully lower the platform of the seesaw (see description of this apparatus below) and eat the food at least 4 times per 15 min session on 2 consecutive sessions over 2 separate training days. Training sessions were performed under natural light cycle with temperatures ranging from 3°C to 25°C.

Once training was completed, the rats were moved outdoors and two colonies of 10 rats were formed. In each colony, there were 2 groups of 5 sisters. The rats were given 7 days to habituate to the enclosures and to each other. The ground of each enclosure was covered with 40 cm of soil, so rats could dig and build tunnels, and the remaining ground was covered with 5 cm of soil (see the subsection “Experimental Subjects and Housing Conditions” of the Methods in the main text for the description of the enclosures). The mean mass of the rats was 283.53 ± 3.54 g, and the mean age of the rats was 469.8 ± 1 days. After the habituation period, we conducted a social learning pilot study from April 20^th^, 2019 to June 15^th^, 2019. Thus, we experimentally introduced innovators in one of the 2 colonies. Five of the 6 rats that were housed with 4 experienced rats successfully manipulated the seesaw to access oats as a food reward, whereas none of the ten rats living without experienced rats successfully accessed the food reward. The pilot study rats were housed indoors after the pilot study in housing cages (see subsection “*Experimental Subjects and Housing Conditions”* in the Methods) until they were euthanized either due to illness or old age.

***S3 Innovations***

In colonies 4 and 5, all rats were naïve (main text Table 2). The first rat to succeed in both colonies without experienced rats likely acquired information by using trial-and-error learning while interacting with the environment and thereby innovated a behaviour that spread through their colony. Four out of 6 rats in colony 4 successfully manipulated the seesaw, and 2 out of 6 rats did so in colony 5 (main text Fig. 2). Most naïve rats in colonies 4 and 5 acquired the use of the seesaw towards the end of the study, so the remaining naïve rats that had not acquired the trait by the end of the study likely may have learned to manipulate the seesaw if there had been more time. Rats also innovated an alternative way to succeed, i.e. by directly lifting the lid to access the food reward, in 4 out of 6 colonies (main text Fig. 5). The first successful manipulation of the seesaw in colony 4 was performed by a rat that directly lifted the lid to access the food reward, and this was the only success by directly lifting the lid out of 133 successes overall in colony 4 (main text Fig. 2, Fig. 5). Hence in this colony, the first rat to succeed innovated a new way to manipulate the seesaw, i.e. directly lifting lid, while the second rat to succeed innovated an alternative way to manipulate the seesaw, i.e. sitting on the platform. In colony 5, 15 of the 19 successful manipulations of the seesaw were direct lifts of the lid (main text Fig. 2, Fig. 5). The first individual to succeed in colony 5 sat on the platform but did not directly lift the lid, while the second individual innovated an alternative way to manipulate the seesaw, by directly lifting the lid. In colony 2, two experienced rats successfully lifted the lid a cumulative total of 3 times (main text Fig. 5). The first rat to directly lift the lid in colony 2 innovated an alternative way to manipulate the seesaw after successfully sitting on the platform 53 times (main text Fig. 5). In colony 6, an experienced rat innovated a new way to manipulate the seesaw, by directly lifting the lid, on its third successful manipulation of the seeaw, and it directly lifted the lid 9 out of 512 times it was successful (main text Fig. 5). One of the naïve rats in this colony accessed the food multiple times by directly lifting the lid, and this rat succeeded twice by sitting on the platform before directly lifting the lid successfully (main text Fig. 2 and Fig. 5).

**Data availability**

We have uploaded the codes and data files (.RData) to the submission. If accepted, we will add the codes and data files to a public repository or as part of the supplemental information. The codes can be opened in R or Rstudio. All the data files are .RData files, which can be opened in R or Rstudio. The Rscript has the codes to open the data files, e.g. load("latency_first_success_naive_rats.RData"). If you have problems opening the data files, please send SCE an email.
